# Analysis of gene expression changes in relation to toxicity and tumorigenesis in the livers of Big Blue transgenic rats fed comfrey (*Symphytum officinale*)

**DOI:** 10.1186/1471-2105-7-S2-S16

**Published:** 2006-09-26

**Authors:** Nan Mei, Lei Guo, Lu Zhang, Leming Shi, Yongming Andrew Sun, Chris Fung, Carrie L Moland, Stacey L Dial, James C Fuscoe, Tao Chen

**Affiliations:** 1 Division of Genetic and Reproductive Toxicology, National Center for Toxicological Research, FDA, Jefferson, AR 72079, USA; 2 Division of Systems Toxicology, National Center for Toxicological Research, FDA, Jefferson, AR 72079, USA; 3 Molecular Biology-SDS/Arrays, Applied Biosystems, Foster City, CA 94404, USA; 4 Solexa, Inc., 25861 Industrial Boulevard, Hayward, CA 94545, USA

## Abstract

**Background:**

Comfrey is consumed by humans as a vegetable and a tea, and has been used as an herbal medicine for more than 2000 years. Comfrey, however, is hepatotoxic in livestock and humans and carcinogenic in experimental animals. Our previous study suggested that comfrey induces liver tumors by a genotoxic mechanism and that the pyrrolizidine alkaloids in the plant are responsible for mutation induction and tumor initiation in rat liver.

**Results:**

In this study, we identified comfrey-induced gene expression profile in the livers of rats. Groups of 6 male transgenic Big Blue rats were fed a basal diet and a diet containing 8% comfrey roots, a dose that resulted in liver tumors in a previous carcinogenicity bioassay. The animals were treated for 12 weeks and sacrificed one day after the final treatment. We used a rat microarray containing 26,857 genes to perform genome-wide gene expression studies. Dietary comfrey resulted in marked changes in liver gene expression, as well as in significant decreases in the body weight and increases in liver mutant frequency. When a two-fold cutoff value and a *P*-value less than 0.01 were selected, 2,726 genes were identified as differentially expressed in comfrey-fed rats compared to control animals. Among these genes, there were 1,617 genes associated by Ingenuity Pathway Analysis with particular functions, and the differentially expressed genes in comfrey-fed rat livers were involved in metabolism, injury of endothelial cells, and liver injury and abnormalities, including liver fibrosis and cancer development.

**Conclusion:**

The gene expression profile provides us a better understanding of underlying mechanisms for comfrey-induced hepatic toxicity. Integration of gene expression changes with known pathological changes can be used to formulate a mechanistic scheme for comfrey-induced liver toxicity and tumorigenesis.

## Background

Comfrey belongs to the family Boraginaceae. Three plant species in the genus *Symphytum *contribute to the crop known as comfrey, *Symphytum officinale *L. (wild or common comfrey; the major comfrey species), *S. asperum *Lepechin (prickly or rough comfrey), and *S. x uplandicum *Nyman (quaker, Russian, or blue comfrey; a natural hybrid of *S. officinale *L. and *S. asperum *Lepechin). *Symphytum officinale *L. is a tall perennial with large hairy leaves and small purple flowers [1]. Comfrey has been used as an herbal medicine for more than two thousand years for the treatment of broken bones, tendon damage, ulcerations in the gastrointestinal tract, and lung congestion, as well as for wound healing and/or reducing joint inflammation when it is applied externally [2].

In addition to essential nutrients, comfrey also contains pyrrolizidine alkaloids (PAs). PAs are constituents of over 6000 plants, and many of them are hepatotoxic and carcinogenic in humans and animals [3]. In the liver, PAs are transformed to pyrroles by the mixed-function oxidases. Pyrroles exert their toxic effect by reacting with cellular macromolecules, including proteins and DNA [4]. Therefore, comfrey's therapeutic use might increase the risk of liver toxicity. Many countries including Canada, Germany, and the UK, have restricted its availability. In 2001, the US Food and Drug Administration requested voluntary compliance for the removal of products containing comfrey [5].

The major hepatotoxic manifestation in humans ingesting comfrey is the hepatic veno-occlusive lesion (VOD) [6,7], also called sinusoidal obstruction syndrome (SOS) [8]. Comfrey is also carcinogenic in rats, which suggests the potential tumorigenic effects of the plant [9]. Hepatocellular adenomas were induced in rats receiving a diet containing comfrey. Feeding rats comfrey leaves produced a dose-dependent reduction in survival and an increase in liver tumor incidence [9]. Comfrey roots are much more toxic than the leaves.

In a previous study, we developed evidence indicating that the liver tumors induced by feeding rats 2% comfrey root were generated by a genotoxic mechanism and that the PAs in the plant were responsible for mutation induction and tumor initiation in rat liver [10]. Considering the fact that rats tolerate diets containing up to 33% comfrey leaves and 8% comfrey roots for relatively long periods of time (at least 6 months) [9], in the present study, we evaluated the mutagenicity of 8% comfrey root. Using a toxicogenomic approach, we analyzed the changes in global gene expressions in the liver of rats following comfrey-treatment.

## Results

### Growth curve of rats fed with 8% comfrey root

**Figure 1:**
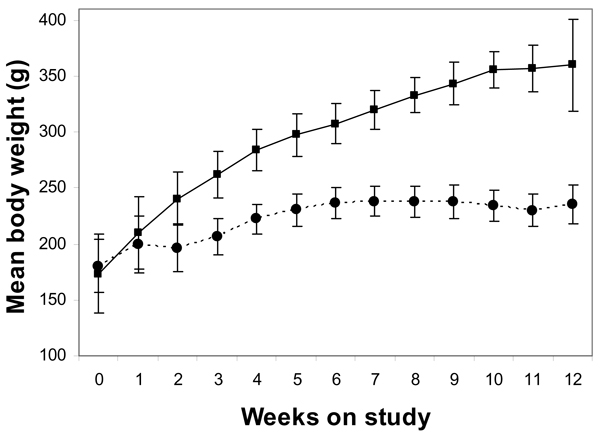
**Mean body weight of Big Blue rats fed with 8% comfrey root for 12 weeks**. The data represent the means ± SD of 6 rats from the control (■) and the 8% comfrey root (●) groups.

Male Big Blue transgenic rats were fed with 8% comfrey root for 12 weeks. The mean body weight of the comfrey-fed rats was less than that of the vehicle controls throughout the study (Figure 1). Rats fed with comfrey weighed 5%, 23%, and 35% less than the control rats after 1, 6, and 12 weeks of the study, respectively, and displayed little weight gain after 6 weeks of feeding with comfrey.

### Mutant frequency (MF) in the liver *cII *gene of comfrey-fed rats

**Figure 2:**
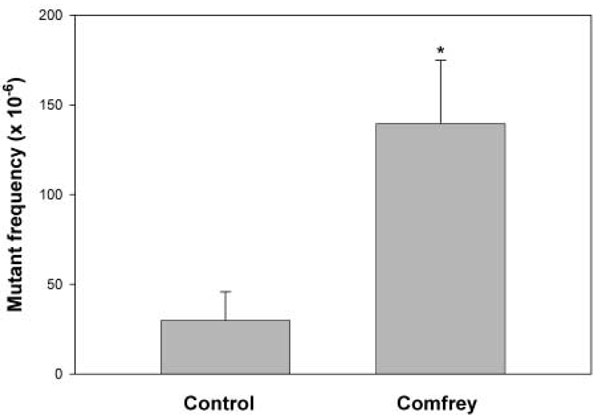
**Liver *cII *mutant frequencies in control and 8% comfrey-fed rats**. The data represent the means ± SD from groups of 6 rats. *Significantly differed from control group (*P *< 0.001).

The results of *cII *MF analyses in the comfrey-fed and control rats are shown in Figure 2. DNA from each liver was packaged 2–4 times either to confirm the MF or to obtain a minimum of 2 × 10^5 ^plaque-forming units for mutant detection. The MF for rats fed with 8% comfrey was 139 ± 35 (SD) × 10^-6^, which was similar to the MF previously detected in 2% comfrey-fed rats [10] and significantly increased over the control group (30 ± 16 × 10^-6^, *P *< 0.001).

### Mutation spectrum in the liver *cII *gene from comfrey-fed rats

Comfrey-induced mutations in the liver *cII *gene were evaluated by DNA sequence analysis of 106 mutants isolated from 6 rats fed a diet containing 8% comfrey root. Since mutations that were found more than once among the mutants isolated from a single animal were assumed to be siblings and to represent only one independent mutation, a total of 99 independent mutations were identified. Table 1 summarizes the types of *cII *mutations observed in the livers of rats fed 8% comfrey compared with mutation spectra of control and 2% comfrey-fed rats that we reported previously [10]. The overall pattern of mutations in 8% comfrey-fed rats differed significantly from controls (*P *< 0.0001), but did not differ from 2% comfrey-fed rats. Among the independent mutations, about 85% from both the comfrey-treated and control rats were base pair substitutions. G:C → T:A transversion (41%) was the major type of mutation in the 8% comfrey-fed rats, whereas G:C → A:T transition was the predominant mutation in the controls. In addition, a 13% frequency of tandem base substitutions was observed among the mutations from the 8% comfrey-fed rats. The 8% comfrey-fed rats had more large deletions than control rats, although the percentages of frameshift mutations were the same.

### Gene expression regulated by comfrey-treatment

**Figure 3:**
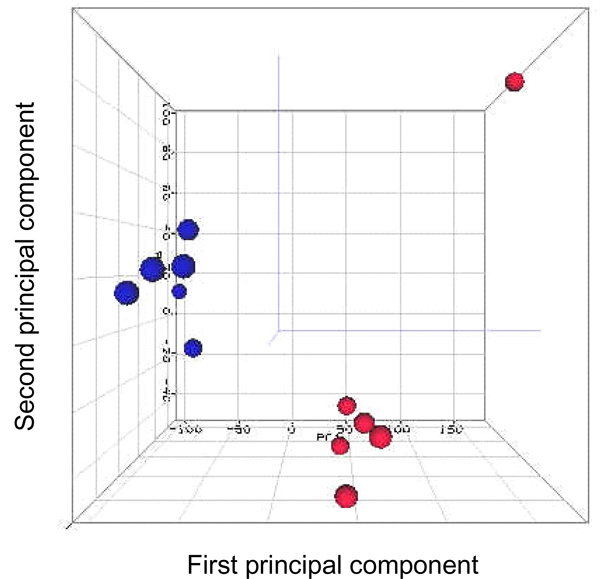
**Principal component analysis for gene expression profiles from livers of control and 8% comfrey-fed rats**. No specific cut off was applied and the intensity of whole rat genome data was used. The red and blue dots indicate control and comfrey-treated samples, respectively.

**Figure 4:**
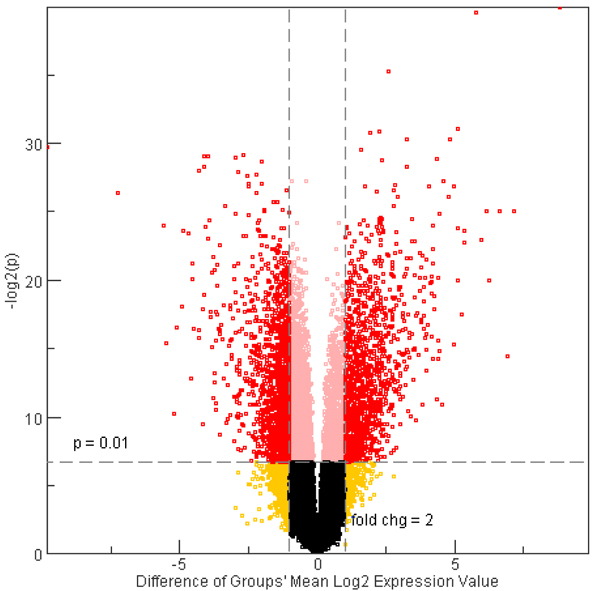
**Volcano plots (log2 fold change vs. -log2 *P*-value)**. A gene was identified as significantly changed if the fold change was greater than 2 (up or down) and the *P*-value was less than 0.01 in comparison to the control group. Each group consisted of 5–6 replicates.

In order to determine gene expression changes associated with comfrey exposure, we employed the Genome Survey Microarray, which contains 26,857 verified rat genes. We isolated total RNA from the livers of 6 control and 6 comfrey-fed rats. For the purpose of visualizing the data, the intensities of the whole rat gene data were analyzed by Principal Components Analysis within ArrayTrack (Figure 3). It demonstrates that one array of sample 6 from the control group appears to be quite different from the rest of the arrays, which was further identified as an outlier array by the Pearson's correlation coefficient of pair-wise log2 intensity correlation (data not shown). This outlier was excluded from further data analysis. A separation between control and comfrey-treated groups was clearly observed, suggesting that there was a clear comfrey-treatment effect on liver gene expression (Figure 3). To select significant genes, minimum requirements were established for both a two-fold change in the gene expression compared to the controls and a *P*-value less than 0.01 for the difference. A total of 2,726 genes satisfied the requirements; 1,235 genes were up-regulated and 1,491 genes were down-regulated in response to comfrey treatment (Figure 4). Among the regulated genes, 1,671 were identified by Ingenuity Pathway Analysis. In this study, we focused on genes involved in metabolism, injury of endothelial cells, and liver injury and abnormalities.

### Genes associated with drug metabolizing genes

Since the PAs in comfrey require metabolic activation to exert their biological effects, we investigated the gene expression changes of drug metabolizing genes. We observed the up- or down-regulation of many cytochrome P450 genes (e.g., Cyp2c12, Cyp3a18, Cyp4a12, Cyp26), glutathione *S*-transferases (Gsta3, Gstm3, and Gstp1), ATP-binding cassette transporters (e.g., Abcb9 and Abcc3), and other metabolism-associated genes, including NAD(P)H oxidoreductase (Nqo1) and aldehyde dehydrogenase (Aldh1a1). Table 2 shows the significant changes in gene expression related to phase I, II, and III drug metabolizing genes.

### Genes involved in injury of endothelial cells

Considering the involvement of sinusoidal endothelial cells and sometimes hepatic venular endothelial cells in VOD/SOS [8], we focused on genes expressed in endothelial cells. Table 3 summarizes the alteration of genes related to cell death, apoptosis, and cell growth of endothelial cells. For example, we observed the up-regulation of previously reported tumor necrosis factors (Tnfsf10, Tnfrsf6, and Tnfrsf12a), transforming growth factor (Tgfb1 and Tgfb2), and other genes (such as, Mmp2, PAI-1 or Serpine1, Plaur, Col4a2, and Edn1) [8,11,12]. Several novel genes were responsive to comfrey treatment, including activating transcription factor 3 (Atf3), B-cell cLL/lymphoma 2 (Bcl2), caspase 1 (Casp1), mitogen-activated protein kinase 9 (Mapk9), and secreted phosphoprotein 1 (Spp1).

### Genes involved in liver injury and abnormalities

Endothelial damage can lead to hepatocellular injury, and fibrotic reaction in the sinusoids is characterized as the later stages of VOD/SOS. Chronic comfrey treatment also resulted in the induction of a number of genes involved in liver injury and abnormalities. Significantly changed genes were divided into subsets based on functionality, and categories included cell death, growth, fibrosis, morphology, and liver cancer development (Table 4). Sixteen genes were functionally categorized in liver cell death. Besides the tumor necrosis factors and transforming growth factor, comfrey treatment resulted in strong up-regulation of cyclin-dependent kinase inhibitor 1A (Cdkn1a) and heme oxyenase 1 (Hmox1), as well as down-regulation of epidermal growth factor (Egf) and CCAAT/enhancer binding protein beta (Cebpb). Of note, genes involved in cell growth were up-regulated along with the genes involved in liver cell death, such as cyclin E1 (Ccne1), hepatocyte growth factor (Hgf), and tissue inhibitor of metalloproteinase 1 (Timp1).

Liver fibrosis is the hallmark of all chronic liver diseases, irrespective of their origin. Ingenuity Pathway Analysis showed that sixteen significantly regulated genes appeared to have a role in liver fibrosis (Table 4). Most of them encode cytokines (Hgf, Igf1, Lif, Tgfb1, and Tnfrsf6), angiogenic molecules (Agt, and Agtr1a), chemokines and their receptors (Ccr2, Cxcl4, and Cxcl12), coagulation cascade genes (PAI-1/Serpine1 and F11), and cytoskeletal molecules (Krt2-8). Moreover, eight genes related to the development of liver cancer were among the most discriminating genes involved in cancer development, such as growth arrest and DNA damage inducible 45 alpha (Gadd45a), B-cell cLL/lymphoma 2 (Bcl2), N-myc downstream regulated 1 (Ndr1), and xeroderma pigmentosum complementation group C (Xpc).

## Discussion

Comfrey was one of the most popular herbal teas in the world, including the United States. Although its popularity has declined due to the understanding of its dangers, it is still available commercially in several forms. The regular use of comfrey is a potential health risk owing to the presence of PAs. Comfrey contains as many as nine PAs, including acetyl intermedine, acetyl lycopsamine, echimidine, intermedine, lasiocarpine, lycopsamine, symlandine, symphytine, and symviridine [13-15]. The PA content of comfrey is less than 1% and depends on the plant part [15]. Higher PA concentrations occur in the roots of comfrey than in the leaves, and commercially available comfrey tablets containing high levels of PAs are likely to be derived from comfrey roots [16,17]. PAs are the leading plant toxins associated with disease in humans and animals.

Several cases of VOD/SOS associated with comfrey ingestion have been reported in humans [6,7,18-20], as well as in experimental animals [21]. Deleve and her colleagues [22] have developed a reproducible animal model of hepatic VOD, in which the rats are gavaged with a single dose of PA, monocrotaline. The model exhibits the characteristic clinical and histological features of hepatic VOD, including the earliest manifestations (progressive injury to the sinusoidal endothelial cells and central vein endothelium), early VOD (centrilobular coagulative necrosis and severe sinusoidal injury, hemorrhage, and central vein endothelial damage), and late VOD (fibrotic occulusion of the central veins) [22]. Comfrey and the specific alkaloids in comfrey (e.g., symphytine and lasiocarpine) induce hepatoadenoma and hemangioendothelial sarcomas in rats [9,23,24]. The mechanisms by which toxicity and carcinogenicity are produced are still not fully understood. Gene expression profiling offers a powerful approach for identifying differentially expressed genes and identifying mechanisms.

Gene expression was markedly affected (Figure 3) in the livers of rats exposed to 8% comfrey root, a dose that resulted in significant decreases in body weight (Figure 1) and increases in liver MF (Figure 2). Out of 26,857 genes evaluated, the expression of 4,132 (15%) and 9,937 (37%) genes were altered more than 2-fold and 1.5-fold, respectively. At *P*-values of 0.01 and 0.05, 7,518 (28% of expressed genes) and 1,0341 (39%) genes, respectively, displayed a significant effect after comfrey-treatment compared to control group. In this study, differential gene expression was considered significant for genes showing at least a 2-fold up- or down-change, and a *P *< 0.01. In total, 2,726 genes (10%) satisfied the requirements and about half of them were down-regulated and half up-regulated in response to comfrey exposure (Figure 4). Such a large number of significantly altered genes may partly reflect the therapeutic effects of comfrey exerted through plant components other than PAs [15]. In the present study, we concentrated on the analysis of genes involved in metabolism, injury of endothelial cells, and liver injury and abnormalities.

Liver is the major organ for biotransformation of xenobiotics and drugs. PAs are metabolically activated to toxic, alkylating pyrroles by mixed-function oxidases. The cytochrome P450 (Cyp) superfamily contains 57 genes and plays a critical role in the phase I metabolism of a variety of xenobiotics including drugs, carcinogens, steroids and eicosanoids [25]. In the present study, comfrey exposure resulted in changes in the expression of 19 Cyp genes (Table 2). Among phase I, II, and III drug metabolizing genes, Cyp2c12, Cyp7a1, Cyp26, Gsta3, and Abcc3 were increased 6-21-fold. In contrast, Cyp2c, Cyp39a1, Gstm3, Abcc8, and others were reduced in the comfrey-treated livers. It is known that many herbal/dietary constituents form reactive intermediates capable of irreversibly inhibiting various Cyps (reviewed in [25]). The resultant metabolites lead to Cyp inactivation by chemical modification of the heme, the apoprotein, or both, as a result of covalent binding of modified heme to the apoprotein. Phase II consists of conjugating enzymes, such as glutathione *S*-transferases (GSTs), UDP-glucuronosyltransferases (UGTs), and sulfatases. GSTs also participate in oxidative stress release pathways. The altered expression of phase I and phase II enzymes along with altered drug transport proteins (phase III) could contribute to the increased susceptibility of rats to carcinogenic chemicals, such as comfrey.

Sinusoidal endothelial cells are more susceptible than hepatocytes to PAs that cause VOD/SOS [22]. Functional annotation extracted from Ingenuity Pathway Analysis revealed that many of the transcriptional responses were associated with the apoptosis, cell death, adhesion, and cell movement of endothelial cells (Table 3). The genes in these pathways were highly expressed in comfrey-treated livers; these genes included the endothelial cell markers plasminogen activator inhibitor type 1 (PAI-1, also known as Serpine1 and Serpine2) and tissue plasminogen activator (Plat), and the cytokine tumor necrosis factors (Tnfrsf6, Tnfrsf12a, and Tnfsf10), and transforming growth factors (Tgfb1 and Tgfb2). Induction of a number of genes involved in the injury of endothelial cells was also detected, including endothelin 1 (Edn1), urokinase plasminogen activator receptor (Plaur), collagen type IV alpha 2 (Col4a2), matrix metalloproteinase 2 (Mmp2), mitogen-activated protein kinase 9 (Mapk9), and secreted phosphoprotein 1 (Spp1). It has been reported that Edn1 is a mediator of hepatic sinusoidal constriction, and increased activity of matrix metalloproteinases is responsible for changes of sinusoidal endothelial cells [11]. Elevated plasma PAI-1 levels are useful in distinguishing VOD/SOS [26]. Endothelial injury is the initiating event in the cascade of events leading to the hepatic changes and clinical manifestation of VOD/SOS [27]. Our results offer a more comprehensive overview of the molecular responses to comfrey exposure by expression of multiple genes in liver endothelial cells.

PAs in comfrey can reach the hepatocytes via the sinusoidal blood, and their toxic metabolites lead to immediate damage to the hepatocytes [4]. Genes involved in liver cell death and growth were also induced or repressed in response to comfrey treatment (Table 4), including tumor necrosis factors, transforming growth factor β, chemokine receptor CCR2 gene (Ccr2), heme oxygenase 1 (Hmox1), immediate early response 3 (Ier3), cyclin-dependent kinase inhibitor (Cdkn1a), inhibin beta A (Inhba), and tissue inhibitor of metalloproteinase 1 (Timp1). It is well known that Hmox1 induction is a protective mechanism against the oxidative stress associated with liver injury [28], and that elevated Hgf could protect hepatocytes from injury or promote hepatocellular regeneration [29]. Since Inhba, so-called activin A, is a negative regulator of hepatocyte cell growth [30], the decreased expression of Inhba observed after comfrey treatment suggests the induction of hepatic growth.

Necrosis of hepatocytes and mesenchymal cells follows comfrey-induced liver cell injury, and functional cells are replaced by fibrotic tissues [27,31,32]. In the present study, we observed 16 genes involved in the function of liver fibrosis by Ingenuity Pathway Analysis (Table 4). Liver fibrosis is characterized by cell proliferation and the accumulation of extracellular matrix components and is mediated by cytokines and growth factors, of which TGF-β1 appears to be a key mediator [32]. The up-regulation of cytokines Hgf and Tnfrsf6 and down-regulation of Igf1 and Lif play an important role in the pathogenesis of liver injury and fibrosis. Decreased serum Igf-1 levels provide a useful index of hepatocellular dysfunction and impaired nutritional status, and increased Hgf appears to limit liver fibrosis [33]. Agt and Agtr1a, cytokines with vasoactive properties, also regulate liver fibrogenesis. Chemokines have a much wider biological role including angiogenesis, carcinogenesis, and cell cycle control [34]. Chemokines in the liver (Cxcl4 and Cxcl12) may modulate the progression of liver fibrosis through their actions on hepatic stellate cells.

PA-induced DNA damage in the liver (endothelial cells and hepatocytes), if not repaired prior to DNA synthesis, might produce replication errors and mutations, which eventually could result in the development of neoplasmas in the treated animals. We determined MFs in the liver *cII *gene of Big Blue transgenic rats. After feeding with 8% comfrey root for 12 weeks, we observed a 4-fold higher MF in the liver *cII *gene compared to the controls (Figure 2). The induction of mutation was similar to that reported previously for rats fed with 2% comfrey root [10]. These observations suggest that the rats could not tolerate the feeding of roots in concentrations over 2%, in terms of mutation induction. Furthermore, the overall pattern of mutations induced by 8% comfrey in liver was similar to that in the livers of rats fed 2% comfrey root (Table 1), whereas both the 2% and 8% comfrey-induced mutation spectra were significantly different from liver controls. In contrast to the G:C → A:T transition that was the predominant mutation in the controls, the major type of mutation in the 8% comfrey-fed rats was G:C → T:A transversion (41%), a mutation that was also induced by riddelliine, a representative genotoxic PA that is tumorigenic for rat liver [35]. In addition, 13% of mutations from the 8% comfrey-fed rats were tandem base substitution, which has been suggested as a mutational signature for the genetic damage of PAs [36]. G:C → T:A transversion may cause the initiation of tumors in the liver of rats fed with comfrey, because it has been reported that more than half of riddelliine-induced liver hemangiosarcomas have a G → T mutation at K-*ras *codon 12 [37]. *p53 *mutation also has been detected at an early stage of riddelliine exposure [38]. Mutations are thought to be involved in carcinogenesis because the transition from a normal somatic cell to a cancer cell is due to mutations in protooncogenes, tumor suppressor genes and/or genes that function in the maintenance of genomic stability [39,40]. Comfrey-induced neoplasms in the rat were mostly found in the liver. Hepatocellular adenomas were induced in all experimental groups that received diets containing 1–8% comfrey root or 8–33% comfrey leaves. In addition, a few rats bearing hepatocellular adenomas simultaneously had hemangioendothelial sarcoma of the liver [9]. The first hepatocellular carcinoma (HCC) appearance was 7 months after initiating the 8% comfrey diet.

Ingenuity Pathway Analysis found 8 genes (2 down- and 6 up -regulated) involved in liver cancer development that were altered due to comfrey treatment (Table 5). In mammals, the nucleotide excision repair process is the most important repair pathway for elimination of DNA damage caused by exogenous agents, including UV light, DNA-reactive carcinogens, and some endogenously generated oxidative lesions [41]. Xeroderma pigmentosum group C (Xpc) is implicated in the early steps of this repair pathway. A significantly higher incidence of chemically induced liver and lung tumors is observed in Xpc null mice [42]. Gap junction membrane channel protein beta 1 (Gjb1), also called connexin 32 (Cx32), is the main gap junction protein in hepatocytes and plays an important role in the regulation of signal transfer and growth control in the liver. It has been reported that Cx32 expression decreases gradually as liver disease progresses to cirrhosis and HCC [43], and a low expression of Cx32 mRNAs in HCC tissues is also predictive of the postoperative recurrence of HCCs [44]. Bcl-2 is characterized as an antiapoptotic/oncogenic protein and also functions as an antioxidant. Increased Bcl-2 expression in cirrhotic patients correlates with the development of HCC [45]. Bcl-2 is also expressed in HCC tissues and the increasing Bcl-2 expression associated with HCC progression suggests that the Bcl-2 protein takes part in the formation of HCC [46]. Hgf, identified originally as the most potent mitogen for hepatocytes, is now known to be a cytokine with numerous functions in a wide variety of cells [47]. It is up-regulated in inflammatory liver diseases and stimulates DNA synthesis preferentially in initiated hepatocytes, presumably resulting in tumour promotion [48]. N-myc downstream-regulated gene 1 (Ndr1, or Ndrg1) plays a role in growth arrest and cell differentiation, is induced by several stress conditions, and is overexpressed in many cancers [49]. Expression of Ndrg1 was significantly up-regulated in HCC tissues compared to that of noncancerous and normal liver tissues [50]. Cyclin E (Ccne1), a regulatory subunit of cyclin-dependent kinase 2, is an important regulator for entry into the *S *phase of the mammalian cell cycle. Overexpression of Ccne1 has been observed in many tumors including primary HCCs [51]; overexpression results in chromosome instability and thus may contribute to tumorigenesis [52]. Growth arrest and DNA damage 45 alpha (Gadd45a) is a nuclear protein involved in the maintenance of genomic stability, DNA repair, and the suppression of cell growth [53]. Gadd45a protein levels are higher in liver cirrhotic and neoplastic tissues [54]. Tissue inhibitor of metalloproteinase 1 (Timp1) is a contributory factor to fibrosis of a variety of organs including the liver. Timp1 and other extracellular matrix remodeling genes are implicated in the transition from mild to moderate fibrosis in patients with chronic hepatitis C [55].

## Conclusion

**Figure 5:**
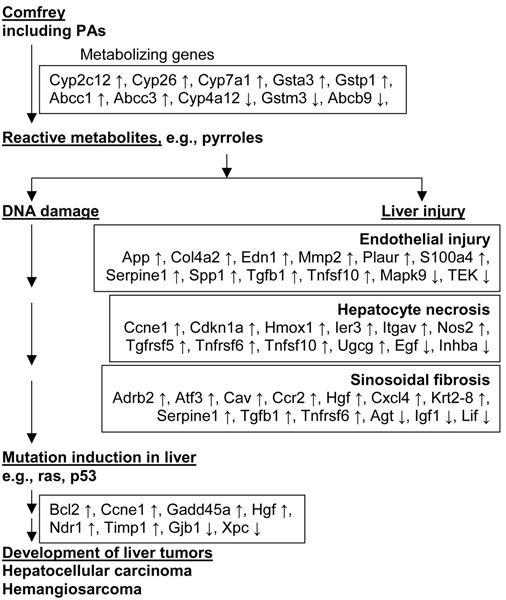
Integration of gene expression with the proposed mechanistic pathway initiated by comfrey treatment leading to tumorigenesis. Responses in boxes represent gene expression observations and arrows in the boxes indicate direction of indicated response.

The integration of gene expression changes with a mechanistic pathway analysis suggests a scheme for comfrey treatment leading to tumorigenesis via mutation induction (Figure 5). The available evidence suggests that active metabolites of PAs in comfrey interact with endothelial and hepatocyte DNA, causing damage to hepatic endothelial cells and hepatocytes. This may result in liver fibrosis and increases in mutation induction, which may be associated with the development of HCC and hemagiosarcoma. We have identified 2,726 genes in the livers of comfrey-fed rats that were differentially expressed. Some of the gene changes are associated with the metabolism, injury of endothelial cells, and liver injury and abnormalities that are postulated to occur as a result of comfrey exposure. This approach provides further insight into the mechanisms involved in the development of VOD/SOS and tumorigenesis after exposure to comfrey.

## Materials and methods

### Plant material and animals

Comfrey roots (*Symphytum officinale*) were purchased from Camas Prairie Products (Trout Lake, WA). Male Big Blue Fisher 344 transgenic rats were obtained from Taconic Laboratories (Germantown, NY) through purchase from Stratagene (La Jolla, CA). All animal procedures followed the recommendations of the NCTR Institutional Animal Care and Use Committee for the handling, maintenance, treatment, and sacrifice of the rats.

### Comfrey diet and treatments

The comfrey roots were ground into powder using a Wiley Mill, and the comfrey root powder was stored at room temperature until use. NIH-31 pellets (Purina Mills International, Brentwood, MO) were autoclaved and ground into meal form. The base diet was blended with comfrey root powder in a Hobart Mixer to make 8% comfrey root dosed diet, which was provided in rat feeders. The treatment schedule was based on the protocol used in a carcinogenesis assay [9]. Male, 6-week-old Big Blue rats were fed without (vehicle control group) or with 8% comfrey roots for 12 weeks. Six rats from each treatment group were sacrificed at the end of the treatment. The livers were isolated, frozen quickly in liquid nitrogen, and stored at -80°C.

### *cII *mutation assay

High-molecular-weight genomic DNA was extracted from rat livers using the RecoverEase DNA Isolation Kit (Stratagene) and stored at 4°C until DNA packaging was performed. The packaging of the phage, plating the packaged DNA samples, and determination of MF were carried out following the manufacturer's instructions for the λ Select-*cII *Mutation Detection System for Big Blue Rodents (Stratagene). The shuttle vector containing the *cII *target gene was rescued from total genomic DNA with phage packaging extract (Transpack, Stratagene). The plating was performed with the *Escherichia coli *host strain G1250. To determine the total titer of packaged phages, G1250 bacteria were mixed with 1:3000 dilutions of phage, plated on TB1 plates, and incubated overnight at 37°C (nonselective conditions). For mutant selection, the packaged phages were mixed with G1250, plated on TB1 plates, and incubated at 24°C for about 42 h (conditions for *cII*-selection). Under these conditions, phages with wild-type *cII *genes undergo lysogenization and become part of the developing bacterial lawn, whereas phages with mutated *cII *genes undergo lytic growth and give rise to plaques. When incubated at 37°C, phages with wild-type *cII *genes also undergo a lytic cycle, resulting in plaque formation. Assays were repeated until a minimum of 2 × 10^5 ^plaque-forming units from each sample were examined for mutation. The *cII *MF is defined as the total number of mutant plaques (determined at 24°C) divided by the total number of plaques screened (determined at 37°C).

### Sequence analysis of the *cII *mutants

The mutants were sequenced according to the method of Mei et al. [36]. The *cII *mutant plaques were selected at random from different animals and replated at low density to verify the mutant phenotype. Single, well-isolated plaques were selected from these plates and transferred to a microcentrifuge tube containing 100 μl of sterile distilled water. The tube was heated at 100°C for 5 min and centrifuged at 12,000 *g *for 3 min. The *cII *target DNA for sequencing was amplified by PCR using primers 5'-AAAAAGGGCATCAAATTAACC-3' (upstream) and 5'-CCGAAGTTGAGTATTTTTGCTG-3' (downstream). For PCR amplification, 10 μl of the supernatant were added to 10 μl of a PCR Master Mix (Promega, Madison, WI) and the primers. The final concentrations of the reagents were: 1× Taq polymerase reaction buffer, 0.2 μM of each primer, 200 μM of each dNTP, 1.5 mM MgCl_2_, and 0.25 U of Taq DNA polymerase. The PCR reactionwas performed using a PCR System 9700 (Applied Biosystems, Foster City, CA), with the following cycling parameters: a 3 min denaturation at 95°C, followed by 35 cycles of 30 s at 95°C, 1 min at 60°C, and 1 min at 72°C, with a final extension of 10 min at 72°C. The PCR products were isolated using a PCR purification kit (Qiagen, Chatsworth, CA). The *cII *mutant DNA was sequenced with a CEQ Dye Terminator Cycle Sequencing Kit and a CEQ 8000 Genetic Analysis System (Beckman Coulter, Fullerton, CA). The primer for *cII *mutation sequencing was the upstream primer used for the PCR.

### Statistical analyses for mutagenicity data

Analyses were performed using the SigmaStat 2.03 program (SPSS, Chicago, IL). All of the MF data were expressed as the mean ± standard deviation (SD) from 6 rats per group. Statistical significance was determined by one-way analysis of variance (ANOVA) followed by the Holm-Sidak test. Mutational spectra were compared using the computer program written by Cariello and colleagues [56] for the Monte Carlo analysis developed by Adams and Skopek [57].

### RNA isolation and quality control

Total RNA was isolated from liver tissues of 6 control and 6 comfrey-fed rats using an RNeasy system (Qiagen). The yield of the extracted RNA was determined spectrophotometrically by measuring the optical density at 260 nm. The purity and quality of extracted RNA were evaluated using the RNA 6000 LabChip and Agilent 2100 Bioanalyzer (Agilent Technologies, Palo Alto, CA). RNA samples with RNA integrity numbers (RINs) greater than 7.5 were used for microarray experiments performed using Applied Biosystems' Rat Genome Survey Microarray platform, which is a one channel microarray with chemiluminescence detection, and contains 26,857 probes (60-mer) for the interrogation of 27,088 genes and 1592 controls that track system performance through each experiment.

### Preparation of digoxigenin labeled in vitro transcribed cRNA

All RNA targets were labeled using the Applied Biosystems RT-IVT Labeling Kit Version 2.0. Briefly, 1.5 μg of total RNA was reverse transcribed via 2 h incubation at 42°C with ArrayScript RT enzyme (Ambion, Austin, TX) and oligo dT-T7 primer. Double stranded cDNA was produced following 2 h incubation with *E. coli *DNA polymerase and RNase H at 16°C. Double-stranded cDNA was purified according to the RT-IVT kit protocol. In vitro transcription was performed by incubation of the cDNA product with T7 RNA polymerase, 0.75 mM Digoxigenin-11-UTP (Roche Applied Science, Indianapolis, IN) and all other NTPs for 9 h. Labeled cRNA was purified according to the RT-IVT kit protocol and analyzed for quality and quantity using standard UV spectrometry and the Bioanalyzer.

### Hybridization of labeled cRNA to microarrays and microarray imaging

Digoxigenin labeled cRNA targets were hybridized to Applied Biosystems Rat Whole Genome Survey Microarrays using the Applied Biosystems Chemiluminescent Detection Kit. Briefly, 15 μg of labeled cRNA targets were fragmented via incubation with fragmentation buffer provided in the kit for 30 min at 60°C. Fragmented targets were hybridized to microarrays during a 16 h incubation at 55°C with buffers and reagents from the Chemiluminescent Detection Kit. Post-hybridization washes and anti-Digoxigenin-Alkaline Phosphatase binding were performed according to the protocol of the kit. Chemiluminescence detection, image acquisition and analysis were performed using Applied Biosystems Chemiluminescence Detection Kit and Applied Biosystems 1700 Chemiluminescent Microarray Analyzer following the manufacturer's protocols. Images were auto-gridded and the chemiluminescent signals were quantified, corrected for background, and finally, spot- and spatially-normalized using the Applied Biosystems 1700 Chemiluminescent Microarray Analyzer software version 1.1.

### Microarray data analysis

Gene expression data from the Applied Biosystems' Rat Genome Survey Microarray were input to ArrayTrack, a software system developed by the FDA's National Center for Toxicological Research for the management, analysis, visualization and interpretation of microarray data [58]. Raw microarray intensity data were normalized per chip to the same median intensity value of 500. Chemiluminescent signals from 1529 control probes that track system performance through each experiment were not used in normalization. The identification of differentially expressed genes based on *t*-tests and fold-change cutoffs, and Principal Component Analysis were conducted within ArrayTrack. Ingenutity Pathway Analysis (Mountain View, CA) was used for pathway and function analysis.

## Authors' contributions

NM performed the animal treatment and mutagenicity analysis, was involved in the analysis of microarray data, and wrote the manuscript. LG, LS, and JCF helped conceive the experiments, analysis of the data, and writing of the manuscript. LZ, YAS, and CF conducted the microarray experiment and generated the raw data. CLM and SLD performed technical support. TC had the original idea for this study, and was involved in designing the experiment and writing the manuscript. All authors approved the final version of manuscript.

## Figures and Tables

**Table 1:** Summary of independent mutations in the liver *cII *gene from comfrey-fed and control Big Blue rats

**Type of mutation**	Control *		**8% comfrey **•		**2% comfrey **•, *	
	
	**Number**	**%**	**Number**	**%**	**Number**	**%**
G:C → C:G	5	11	6	6	11	6
G:C → A:T	20	43	11	11	24	12
G:C → T:A	9	20	40	41	83	42
A:T → T:A	1	2	4	4	5	2
A:T → G:C	1	2	6	6	9	4
A:T → C:G	3	7	4	4	7	3
Frameshift	7	15	14	14	26	13
Complex	0	0	1	1	2	1
Tandem-base substitution	0	0	13	13	33	17
Total mutants screened	46	100	99	100	200	100

•Spectra for 2% and 8% comfrey-fed rats were significantly different from the controls (*P *< 0.001). There was no significant difference between the spectra for 2% and 8% comfrey.

*Data are from literature [10].

**Table 2:** Genes involved in drug metabolism altered by comfrey treatment in liver

**Gene symbol**	**Gene description**	**Locus link ID**	**Fold change**	***P*-value**
*Phase I metabolism*				
Cyp2c	cytochrome P450, family 2, subfamily c	29277	0.03	0.00000
Cyp2c12	cytochrome P450, family 2, subfamily c	25011	6.52	0.00000
Cyp2c39	cytochrome P450, family 2, subfamily c	29298	0.35	0.00001
Cyp2d1	cytochrome P450, family 2, subfamily d	266684	0.38	0.00000
Cyp2d2	cytochrome P450, family 2, subfamily d	25053	0.43	0.00002
Cyp2d3	cytochrome P450, family 2, subfamily d	24303	0.39	0.00000
Cyp2d5	cytochrome P450, family 2, subfamily d	286963	0.44	0.00000
Cyp3a18	cytochrome P450, family 3, subfamily a	252931	0.38	0.00060
Cyp4a3	cytochrome P450, family 4, subfamily a	298423	0.46	0.00010
Cyp4a12	cytochrome P450, family 4, subfamily a	266674	0.36	0.00015
Cyp4b1	cytochrome P450, family 4, subfamily b	24307	0.32	0.00008
Cyp4f5	cytochrome P450, family 4, subfamily f	286905	4.71	0.00000
Cyp4f14	cytochrome P450, family 4, subfamily f	56266	0.48	0.00005
Cyp4f18	cytochrome P450, family 4, subfamily f	72054	0.47	0.00014
Cyp7a1	cytochrome P450, family 7, subfamily a	25428	6.16	0.00067
Cyp7b1	cytochrome P450, family 7, subfamily b	13123	2.15	0.00169
Cyp11b3	cytochrome P450, family 11, subfamily b	353498	0.43	0.00032
Cyp26	cytochrome P450, family 26	154985	18.54	0.00005
Cyp39a1	cytochrome P450, family 39, subfamily a	56050	0.29	0.00315
*Phase II metabolism*				
Gsta3	glutathione S-transferase, alpha 3	14859	14.47	0.00001
Gstm3	glutathione S-transferase, mu 3	81869	0.39	0.00173
Gstp1	glutathione S-transferase, pi 1	14869	3.16	0.00001
*Phase III metabolism*				
Abcb9	ATP-binging cassette, subfamily b (MDR/TAP)	63886	0.23	0.00292
Abcc1	ATP-binging cassette, subfamily c (CFTR/MRP)	24565	2.65	0.00132
Abcc3	ATP-binging cassette, subfamily c (CFTR/MRP)	140668	21.20	0.00006
Abcc6	ATP-binging cassette, subfamily c (CFTR/MRP)	81642	0.42	0.00000
Abcc8	ATP-binging cassette, subfamily c (CFTR/MRP)	25559	0.10	0.00000

**Table 3:** Genes involved in endothelial cells altered by comfrey treatment in liver

**Gene symbol**	**Gene description**	**Locus link ID**	**Fold change**	***P*-value**
*Cell death and apoptosis*				
*, • Angpt2	angiopoietin 2	11601	3.45	0.00121
App	amyloid beta (A4) precursor protein	54226	4.69	0.00000
Atf3	activating transcription factor 3	25389	6.22	0.00010
Bcl2	B-cell cLL/lymphoma 2	24224	2.41	0.00500
Bcl2a1	Bcl2-related protein A1	170929	3.43	0.00022
Bcl2l	Bcl2-like	12048	2.53	0.00000
Casp1	caspase 1, apoptosis-related cysteine protease (interleukin 1, beta, convertase)	25166	2.26	0.00551
Cnp	2',3'-cyclic nucleotide 3' phosphodiesterase	1267	2.37	0.00010
• Col4a2	collagen, type IV, alpha 2	1284	2.81	0.00000
• Cxcr4	chemokine receptor (LCR1)	60628	3.22	0.00186
Dusp6	dual specificity phosphatase 6	116663	2.31	0.00036
Edn1	endothelin 1	24323	4.87	0.00033
Fgf1	fibroblast growth factor 1	25317	2.05	0.00057
*Gpr9	G protein-coupled receptor 9 (Cxcr3)	84475	2.02	0.00944
• Hgf	hepatocyte growth factor	24446	3.18	0.00065
Hmox1	heme oxygenase 1	24451	3.23	0.00064
Il2	interleukin 2	116562	0.29	0.00010
Ins1	insulin 1	24505	0.22	0.00000
Lrp5	low density lipoprotein receptor-related protein 5	16973	0.35	0.00010
Mapk9	mitogen-activated protein kinase 9	50658	0.49	0.00009
Pparg	peroxisome proliferator activated receptor, gamma	25664	0.32	0.00033
Serpinf1	serine (or cysteine) proteinase inhibitor, clade F), member 1	287526	0.49	0.00009
*Spp1	secreted phosphoprotein 1	25353	11.63	0.00003
*, • Tgfb1	transforming growth factor, beta 1	59086	2.11	0.00002
Tnfrsf6	tumor necrosis factor receptor superfamily, member 6	246097	3.40	0.00007
Tnfsf10	tumor necrosis factor (ligand) superfamily, member 10	246775	2.19	0.00366
*Adhesion*				
Anxa1	annexin 1	25380	2.50	0.00009
Anxa5	annexin 5	25673	4.29	0.00000
Cd44	CD44 antigen	25406	2.65	0.00004
Cx3cl1	chemokine (C-X3-C motif) ligand 1	89808	2.55	0.00333
• Cxcl4	chemokine (C-X-C motif) ligand 4	56744	3.12	0.00008
Cyr61	cysteine rich protein 61	83476	4.54	0.00000
•Itga4	integrin alpha 4	16401	5.28	0.00002
•Itgav	integrin alpha V	16410	2.00	0.00113
Selplg	selectin P ligand	6404	2.26	0.00088
*Cell movement*				
Agt	angiotensinogen	24179	0.36	0.00000
Ccl2	chemokine (C-C motif) ligand 2	24770	4.27	0.00026
Cd151	CD151 antigen	64315	2.13	0.00000
Cd9	CD9 antigen	12527	2.10	0.00127
Ceacam1	carcinoembryonic antigen-related cell adhesion molecule 1	81613	0.49	0.00138
Cxcl12	chemokine (C-X-C motif) ligand 12	24772	0.19	0.00000
Edil3	EGF-like repeats and discoidin I-like domains 3	10085	3.57	0.00015
Efnb2	ephrin B2	13642	0.49	0.00006
Fgfr1	Fibroblast growth factor receptor 1	79114	2.25	0.00002
Id1	Inhibitor of DNA binding 1, helix-loop-helix protein (splice variation)	25261	2.89	0.00068
Lgals3	lectin, galactose binding, soluble 3	83781	2.31	0.00003
Mmp2	matrix metalloproteinase 2	17390	4.31	0.00000
Plaur	urokinase plasminogen activator receptor	50692	2.93	0.00164
Ptk2b	protein tyrosine kinase 2 beta	50646	3.85	0.00000
S100a4	S100 calcium-binding protein A4	24615	3.61	0.00001
Serpine1	serine (or cysteine) proteinase inhibitor, clade E, member 1 (plasminogen activator inhibitor type 1)	24617	30.94	0.00003
Stc1	stanniocalcin 1	81801	3.50	0.00306
TEK	TEK tyrosine kinase, endothelial (venous malformations, multiple cutaneous and mucosal)	7010	0.34	0.00012
Tgfb2	transforming growth factor, beta 2	81809	2.31	0.00458
Tnfrsf12a	tumor necrosis factor receptor superfamily, member 12a	302965	2.91	0.00090

Genes also involved in adhesion* and cell movement• of endothelial cells.

**Table 4:** Genes involved in liver injury and abnormalities altered by comfrey-treatment

**Gene symbol**	**Gene description**	**Locus link ID**	**Fold change**	***P*-value**
*Cell death of liver cells*				
Bcl2	B-cell cLL/lymphoma 2	24224	2.41	0.00500
Bcl2l	Bcl2-like	12048	2.53	0.00000
*Ccr2	chemokine receptor CCR2 gene	60463	2.47	0.00591
Cdkn1a	cyclin-dependent kinase inhibitor 1A (p21)	114851	4.83	0.00014
Cebpb	CCAAT/enhancer binding protein (C/EBP), beta	24253	0.46	0.00000
Egf	epidermal growth factor	25313	0.36	0.00000
Hmox1	heme oxygenase 1	24451	3.23	0.00064
Ier3	immediate early response 3	294235	2.24	0.00002
Itgav	integrin alpha V	16410	2.00	0.00113
*Krt2-8	keratin complex 2, basic, gene 8	25626	2.82	0.00000
Nos2	nitric oxide synthase 2, inducible	24599	3.38	0.00491
*, • Tgfb1	transforming growth factor, beta 1	59086	2.11	0.00002
Tnfrsf5	tumor necrosis factor receptor superfamily, member 5	21939	3.58	0.00009
*, • Tnfrsf6	Tumor necrosis factor receptor superfamily, member 6	246097	3.40	0.00007
Tnfsf10	tumor necrosis factor (ligand) superfamily, member 10	246775	2.19	0.00366
Ugcg	UDP-glucose ceramide glycosyltransferase	83626	2.08	0.00000
*Growth of liver cells*				
Ccne1	cyclin E1	898	7.93	0.00000
Edn1	endothelin 1	24323	4.87	0.00033
*, • Hgf	hepatocyte growth factor	24446	3.18	0.00065
Inhba	inhibin beta-A (activin A)	29200	0.08	0.00000
Tgfb1	transforming growth factor, beta 1	59086	2.11	0.00002
Timp1	tissue inhibitor of metalloproteinase 1	116510	4.25	0.00000
*Liver fibrosis*				
Adrb2	adrenergic receptor, beta 2	24176	3.58	0.00000
Agt	angiotensinogen	24179	0.36	0.00000
Agtr1a	angiotensin II receptor, type 1 (AT1A)	24180	0.38	0.00000
Atf3	activating transcription factor 3	25389	6.22	0.00010
Cav	caveolin	25404	3.15	0.00006
Cxcl4	chemokine (C-X-C motif) ligand 4	56744	3.12	0.00008
Cxcl12	chemokine (C-X-C motif) ligand 12	24772	0.19	0.00000
F11	coagulation factor XI	109821	0.43	0.00000
Igf1	insulin-like growth factor 1	24482	0.48	0.00000
Lif	leukemia inhibitory factor	60584	0.41	0.00215
Serpine1	serine (or cysteine) proteinase inhibitor, clade E member 1 (plasminogen activator inhibitor type 1)	24617	30.94	0.00003
*Liver morphology*				
Ccnd1	cyclin D1	58919	5.65	0.00002
Ins1	insulin 1	24505	0.22	0.00000
Junb	Jun-B oncogene	24517	3.77	0.00001
*Liver cancer development*				
Bcl2	B-cell cLL/lymphoma 2	24224	2.41	0.00500
Ccne1	cyclin E1	898	7.93	0.00000
Gadd45a	growth arrest and DNA-damage-inducible 45 alpha	25112	2.15	0.00326
Gjb1	gap junction membrane channel protein beta 1	29584	0.48	0.00003
Hgf	hepatocyte growth factor	24446	3.18	0.00065
Ndr1	N-myc downstream regulated 1 (Ndrg1)	10397	2.26	0.00020
Timp1	tissue inhibitor of metalloproteinase 1	116510	4.25	0.00000
Xpc	xeroderma pigmentosum, complementation group C	7508	0.42	0.00001

*Five genes also involved in liver fibrosis and • three genes also involved in liver morphology.
